# Selection and validation of reference genes by RT-qPCR for murine cementoblasts in mechanical loading experiments simulating orthodontic forces in vitro

**DOI:** 10.1038/s41598-020-67449-w

**Published:** 2020-07-02

**Authors:** Christian Niederau, Rogerio B. Craveiro, Irma Azraq, Julia Brockhaus, Asisa Bastian, Christian Kirschneck, Michael Wolf

**Affiliations:** 1 Department of Orthodontics, Dental Clinic, University of Aachen, Pauwelsstr. 30, 52074 Aachen, Germany; 2 Department of Orthodontics, University Medical Centre of Regensburg, Regensburg, Germany

**Keywords:** Cell biology, Molecular biology, Rheumatology

## Abstract

Different structures and cell types of the periodontium respond to orthodontic tooth movement (OTM) individually. Cementoblasts (OC/CM) located in the immediate vicinity of the fibroblasts on the cement have found way to the centre of actual research. Here, we identify and validate possible reference genes for OC/CM cells by RT-qPCR with and without static compressive loading. We investigated the suitability of 3 reference genes in an in vitro model of cementoblast cells using four different algorithms (Normfinder, geNorm, comparative delta-C_t_ method and BestKeeper) under different confluences and time. Comparable to our previous publications about reference genes in OTM in rats and human periodontal ligament fibroblasts (hPDLF), *Rpl22* in murine OC/CM cells appears as the least regulated gene so that it represents the most appropriate reference gene. Furthermore, unlike to the expression of our recommended reference genes, the expression of additionally investigated target genes changes with confluence and under loading compression. Based on our findings for future RT-qPCR analyses in OC/CM cells, *Rpl22* or the combination *Rpl22/Tbp* should be favored as reference gene. According to our results, although many publications propose the use of *Gapdh*, it does not seem to be the most suitable approach.

## Introduction

The periodontal ligament (PDL), a component of the periodontal apparatus, represents a microenvironment essential for anchoring teeth, fulfils functions of proprioception and buffering of acting forces and it enables periodontal remodeling allowing for orthodontic tooth movement. During orthodontic treatment, teeth are moved through the alveolar bone with the help of mechanical force. This force initially deflects the tooth inside the alveolar cavity^1^. As a result, the periodontium can be divided into tension and compression areas. An inflammatory reaction associated with bone loss and risk of root resorptions on the compression side^2,3^ and bone formation on the tension side has been detected^4^.

The individual role of different cell types involved in orthodontic tooth movement must be identified and understood in order to gain a better insight into the mechanisms of orthodontic tooth movement for research purposes.

Apart from its important role in anchoring teeth, the PDL contributes to tooth nutrition, homoeostasis and damaged tissue repair^5^. PDL fibroblasts (PDLF), PDL stem cells (PDLSC), osteoblasts, osteoclasts and cementoblasts are important cells in this area. PDLFs are the most common and best studied cell type in investigations of tooth movement^1^.

Cementoblasts are located on the cementum covered root surface. Cementum, produced by cementoblasts all lifelong, plays an important role in anchoring the tooth to the surrounding alveolar bone^6,7^. It represents the key structure for attaching Sharpey’s fibers on the tooth side^8^. These fibers are the unique connecting link between tooth and alveolar bone. The absence of cementum results in loss of attachment, a major problem in periodontal degeneration and disease. For this reason, new cementum formation and restoration of soft tissue attachment to the cementum is one of the major goals of regenerative periodontal therapy and research^9^. Furthermore, cementoblasts are assumed to have an essential protective function on the root surface by protecting it from resorption^2^. It has been shown that root resorption lacunas are regenerated with cementum in absence of the causal stimulus (e.g. orthodontic force)^10,11^.

First RT-qPCR data regarding murine cementoblasts under compressive force have already been published^12–15^. Mechanoreceptors, enabling stressed cells to react to compressive stimuli, are less investigated in OC/CM cells. The ion channel Piezo1 seems to be involved in hPDLF^16^ and in OCCM^13^ regulation. In addition, in previous work we were able to demonstrate the participation of TLR-4 after compression of hPDLF^17^. It is also known that compression leads to increased VEGF-a expression on protein level^18^ as well as to enhanced cytokine expression in hPDLF^19^. Moreover, there are information that induced oxygen partial pressure affects RANKL mRNA upregulation in murine cementoblasts^20^ and in hPDLF^21^. In hPDLF, hypoxia related VEGF mRNA upregulation is published^22^. Considering these findings we already investigated the influence of hypoxia regarding to loading compression experiments with hPDLF with the result, that hypoxic effects appear to play a minor role in the regulation of osteoclastogenesis^23^. These findings need to be verified for cementoblasts. However, a systematic validation of reference genes with focus on the in vitro model of mechanical loading in cementoblasts is missing.

The best characterized murine cementoblasts cells were isolated and immortalized by setting SV40 large T antigen under control of the osteocalcin promotor, being the reason for naming these cells osteocalcin cementoblasts (OC/CM)^24^. In vitro, this procedure only immortalizes cells that express osteocalcin (OCN) and SV40, because preliminary in situ studies showed, that OCN was expressed by cementoblasts during root development, but not by PDL cells. Consequently, when populations are isolated from developing molars using collagenase/trypsin digestion, only cementoblasts, not PDL cells, are immortalized and will survive in culture^24,25^. A primary murine cell line is currently not available.

To simulate the mechanical forces acting in periodontal remodeling in vitro, a static mechanic compression model has been developed^26^. For this purpose, a sterile glass cylinder is placed on the monolayer for simulating the stimulus that a cell of the periodontal microenvironment experiences when being compressed between root and alveolar bone during OTM.

Studies of gene expression observing cells under different conditions play an important role in investigating molecular mechanisms. Quantitative real-time PCR (RT-qPCR) is the gold standard for quantification of gene expression due to the method’s merits concerning specificity, sensitivity, accuracy and reproducibility^27^.

Even with greatest care, variations in the path from sample collection to data acquisition can influence the RT-qPCR statement if normalization is not performed with a suitable reference gene^28^. The reference gene should meet three criteria: (1) the amplification efficiency should be similar to the target genes; (2) a moderate expression level; (3) stable expression under all test conditions^29^.

Furthermore, a well working reference gene in condition ‘A’ may not be suitable in condition ‘B’^30^. There is evidence that a reference gene working for one cell type cannot be adopted for other cell types without checking the requirements above^31^. In addition, it is known that some classic reference genes such as *ß-actin* do not perform adequately in mechanically stressed cells^32,33^. We used these findings as a basis for selecting possible genes for the present investigation.

Possible reference genes were purposely selected based on research already made, and their stability in neighboring cells using comparable experimental conditions. We included 3 potential references in our investigation. First, *Gapdh* (glyceraldehyde-3-phosphate dehydrogenase), a glycolysis enzyme which is frequent in the normalization of RT-qPCR and has also been used in previous experiments with murine cementoblasts^13,15^. Second, *Rpl22* (the ribosomal 60S protein L22), a component of the ribosomal 60S subunit which is proven to be the most stable reference gene in compression experiments with human periodontal fibroblasts. Third, *Tbp* (TATA-binding protein), a transcription factor exhibiting stable gene expression under comparable experimental conditions in human periodontal fibroblasts^34^.

Due to the high proliferation rate of OC/CM cells which significantly increase their confluence, we postulate that gene expression of a suitable reference gene should be independent from different cell densities. We examined the expression of different target genes at 60% and 100% confluence to show the effect of an altered confluence on the gene expression of target genes. The chosen target genes are inflammation and remodeling markers. This allows to investigate their changes during this compression associated inflammatory reaction that is accompanied by bone loss.

In this study, we propose recommendable reference genes for OC/CM cells at different confluences and in an in vitro model of orthodontic tooth movement in order to facilitate reliable research respecting the MIQE guidelines^30^. Our publication should provide as a basis for future RT-qPCR analyses with murine cementoblasts, investigating their role in periodontal microenvironment, delineation to other cells and regulation under mechanical stimulation. In addition, we show that precise monitoring of cell confluence is fundamental for comparable RT-qPCR results with these cells.

## Results

### Selection of primer, amplicon quality and specificity, RT-qPCR efficiencies and expression levels

**Figure 1:**
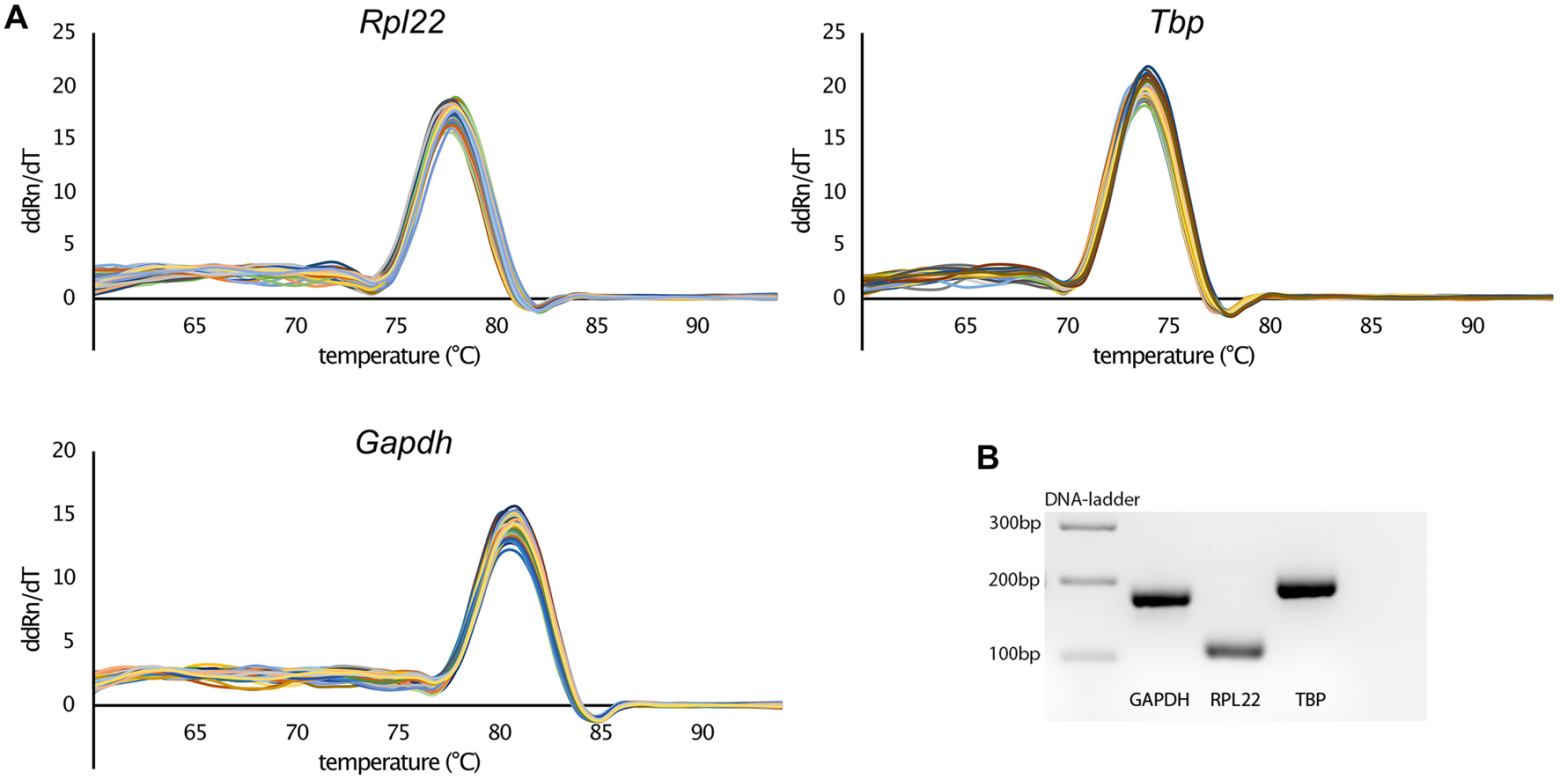
Validation of qPCR primers. (**A**) Melting curve analysis for testing primer specificity. A single peak indicates a single PCR-product. (**B**) qPCR products of each target gene were run on a 2% agarose gel. Each gene shows a single fluorescent band at the expected amplicon size (Table 1). The gel was detected with ChemiDoc MP Imaging System (BioRad), exported as a TIFF image, inverted and cropped to show the relevant gel area. See Supplementary Data 1 for the unedited image. (bp = base pairs).

First of all, we were able to design primers for our candidate reference genes that meet our criteria to ensure adequate PCR specificity and efficiency. Primer specificity was controlled by performing melting curves and gel electrophoresis of the RT-qPCR products. All melting curves show a single peak that indicates the absence of side products and primer dimers (Fig. 1). This is confirmed by electrophoretic separation. For each gene, a single, sharply bordered fluorescent band shows up (Fig. 1, Supplementary Data 1) at the expected molecular amplicon weight (Table 1).

**Table 1:** RT-qPCR gene, primer and target/amplicon information for the 3 investigated candidate references genes and 10 investigated target genes.

Gene symbol	Gene name (*Mus musculus*)	Gene function	Accession number (NCBI Gene Bank)	Chromosoma location (length)	5ʹ-forward primer-3ʹ (length/Tm/%GC)	5ʹ reverse primer-3ʹ (length/Tm/%GC)	Primer location	Amplicon length (bp)	Amplicon location (bp of start/stop)	Intron-flanking (length)	Variants targeted (transcript/ splice)
*Rpl22*	Ribosomal protein L22	Translation of mRNA in protein	NM_001277113.1	4; 4 E2 (2153 bp)	AAGTTCACCCTGGACTGCAC (20 bp/60.18 °C/55%)	AGGTTGCCAGCTTTCCCATT (20 bp/60.18 °C/50%)	Exon 2/3	110	166/275	Yes	Yes
*Tbp*	TATA box binding protein	General transcription factor	NM_013684.3	17 A2; 17 8.95 cM (1842 bp)	GACCCACCAGCAGTTCAGTAG (21 bp/60.34 °C/57.14%)	ATGTGGTCTTCCTGAATCCCTTT (23 bp/59.67 °C/43.48)	Exon 5/7	194	1003/1196	Yes	–
*Gapdh*	Glyceraldehyde-3-phosphate dehydrogenase	Enzyme in glycolysis and gluconeo-genesis	NM_001289726	6 F2; 6 59.32 cM (1296 bp)	CCCCCATGTTTGTGATGGGT (20 bp/60.25 °C/55.25%)	TCTTCTGGGTGGCAGTGATG (20 bp/59.76 °C/55%)	Exon 4/5	177	469/645	Yes	Yes
*IL-6*	Interleukin 6	Important role in bone metabolism; osteoclastogenesis	NM_031168.2	5 B1; 5 15.7 cM (1083 bp)	ACTTCACAAGTCGGAGGCTTA (21 bp/59.03 °C/47.62%)	TTTTCTGCAAGTGCATCATCGT (22 bp/59.45 °C/40.91%)	Exon 2/3	116	220/335	YES	Yes
*IL-1a*	Interleukin 1a	Important role in bone metabolism; osteoclastogenesis	NM_010554	2 F1; 2 62.9 cM (1974 bp)	GCCATTGACCATCTCTCTCTGA (22 bp/59.57 °C/50%)	TGATACTGTCACCCGGCTCT (20 bp/60.32 °C/55%)	Exon 3/4	156	130/285	Yes	Yes
*Spp1 (OPN)*	Osteopontin	Extracellular structural component of bone	NM_001204201.1	5 E5; 5 50.68 cM (1475 bp)	TGGACTGAGGTCAAAGTCTAGGA (23 bp/60.18 °C/47.83%)	ACAGGGATGACATCGAGGGA (20 bp/60.03 °C/55%)	-	126	450/575	–	Yes
*Bglap (OCN)*	Osteocalcin	Marker for bone formation	NM_007541	3 F1; 3 38.82 cM (496 bp)	GGTAGTGAACAGACTCCGGC (20 bp/61.11 °C/60%)	GGGCAGCACAGGTCCTAAAT (20 bp/60.03 °C/55%)	Exon 2/3	177	177/353	Yes	–
*Tnfsf11 (Rankl)*	Tumor necrosis factor (ligand) superfamily, member 11	Differentiation and activation of osteoclasts	NM_011613.3	14 D3; 14 41.26 cM (2243 bp)	CATTTGCACACCTCACCATCAA (22 bp/59.7 °C/45.45%)	CGTTGCTTAACGTCATGTTAGAGAT (25 bp/59.71 °C/40%)	Exon 4/5	120	642/761	Yes	–
*Tnfrsf11b (Opg)*	Tumor necrosis factor receptor superfamily member 11B	Inactivates RANKL	NM_008764.3	15; 15 D1 (2818 bp)	AGACCAGGAAATGGTGAAGAAGAT (24 bp/59.71 °C/41.67%)	CAAGAAGCTGCTCTGTGGTGA (21 bp/60.54 °C/52.38%)	Exon 4/5	104	1023/1126	Yes	–
*Runx2*	Runt related transcription factor 2	Associated with osteoblast differentiation	NM_001145920.2	17 B3; 17 21.33 cM (6475 bp)	TCTCAGTAAGAAGAGCCAGGCA (22 bp/60.82 °C/50%)	TTCGTGGGTTGGAGAAGCG (19 bp/60.3 °C/57.89%)	Exon 6/7	110	2058/2149	Yes	Yes
*Ptgs2 (Cox2)*	Prostaglandin-endoperoxide synthase 2	Involved in prostaglandin synthesis	NM_011198.4	MT (non nuclear) (4460 bp)	TGAGTACCGCAAACGCTTCT (20 bp/59.97 °C/50%)	GCAGGGTACAGTTCCATGACA (21 bp/60 °C/52.38%)	Exon 9/10	126	1543/1668	Yes	–
*Col1a1*	Collagen, type 1, alpha 1	Subunit of the fibril-forming type I collagen	NM_007742	11 59.01 cM 5946 bp	AGCATGACCGATGGATTCCC (20 bp/59.89 °C/55%)	ATTAGGCGCAGGAAGGTCAG (20 bp/59.82 °C/55%)	Exon 48/49	89	4097/4166	Yes	–

*Tm* melting temperature of primer/specific qPCR product (amplicon), *%GC* guanine/cytosine content, *bp* base pairs, *MT* mitochondrial.

Primer efficiency ranges between 90.2 and 100.2% after linear regression with a minimum coefficient of determination R^2^ = 0.9974 and between 96.1 and 106.2% in LineReg (Table2).

**Table 2:** Primer efficiency obtained from a 4 step log_10_ dilution series (100/10/1/0,1 ng/µL) with technical duplicates in 4 (*Tbp*) respectively 5 (*Rpl22*, *Gapdh*) independent probes, logarithmic linearization, linear regression and finally calculated with $E=100*({10}^{-\frac{1}{slope}}-1)$ ^51^.

	Linear regression				LineReg	
	Primer efficiency E_p_ [%] (2^E^/100%)	SD of (2E/100%)	Slope	Coefficient of determination R^2^	Amplification efficiency E_L_ [%] (2^E^/100%)	SD of (2E/100%)
RPL22	99.6 (1.995)	0.027	− 3.334	0.9981	106.2 (2.088)	0.028
TBP	90.2 (1.902)	0.046	− 3.586	0.9984	96.1 (1.946)	0.026
GAPDH	100.2 (2.001)	0.051	− 3.323	0.9974	98.2 (1.975)	0.032

In addition to that, amplification graphs of each well were analyzed with LineReg^41^ (https://www.medischebiologie.nl/files/) to get well-specific efficiencies (n = 50).

**Figure 2:**
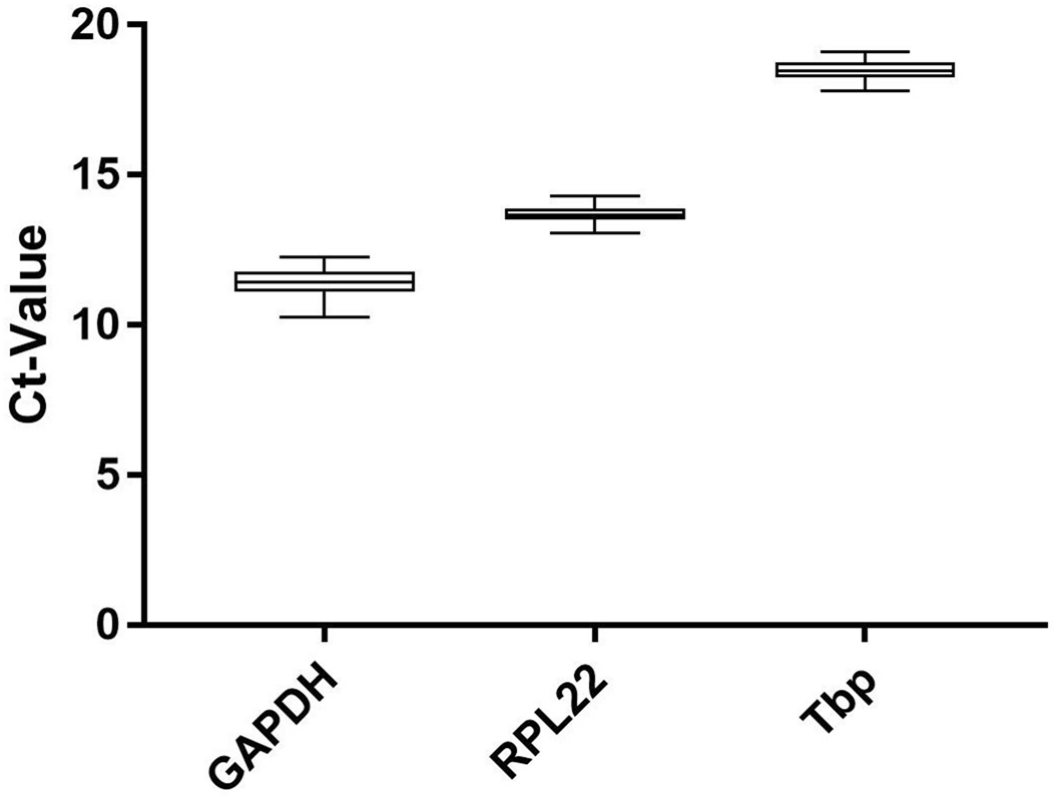
Expression levels of candidate reference genes in all experimental groups (n = 69). C_t_ values exported with identical threshold settings (mean of two technical replicates). Boxplots show median, interquartile range (box) and data range (whiskers).

The average expression of our investigated genes ranges from C_t_ = 11.48 (SD = 0.34; *Gapdh*) to C_t_ = 18.27 (SD = 0.34; *Tbp*) (Fig. 2).

### Stability analysis of candidate reference genes through mathematical algorithms

To test the stability of reference genes we performed analysis with four different algorithms. All 4 algorithms show that *Rpl22* is the least regulated reference gene under loading compression. Although *Tbp* is not as stable as *Rpl22,* it scores better than *Gapdh* in all calculations (Table 3).

**Table 3:** Ranking of reference gene stability for OC/CM cells under compressive orthodontic force (compression vs. control) and controls with different confluences (60%, 70%, 100%).

RefFinder													R			
Total of 4 methods			delta-C_t_		Bestkeeper				Normfinder		geNorm		Normfinder R		geNorm (NormqPCR)	
Ranking order		Rank sum	Ranking order	Stability value	Ranking order	Stability value	SD	CV	Ranking order	Stability value	Ranking order	Stability value	Ranking order	Stability value	Ranking order	Stability value
**OC/CM control + static compression (n = 24)**																
(1)	*Rpl22*	1	*Rpl22*	0.49	*Rpl22*	0.101	1.07	0.78	*Rpl22*	0.086	*Tbp/Rpl22*	0.472	*Rpl22*	0.14	*Rpl22*	0.395
(2)	*Tbp*	1.682	*Tbp*	0.577	*Tbp*	0.263	1.2	1.47	*Tbp*	0.464			*Tbp*	0.24	*Tbp*	0.495
(3)	*Gapdh*	3	*Gapdh*	0.596	*Gapdh*	0.33	1.26	3.03	*Gapdh*	0.501	*Gapdh*	0.554	*Gapdh*	0.37	*Gapdh*	0.576
**OC/CM controls with different confluences (n = 12)**																
(1)	*Rpl22*	1	*Rpl22*	0.439	*Rpl22*	0	1	0	*Rpl22*	0.199	*Rpl22/Gapdh*	0.41	*Rpl22*	0.09	*Rpl22*	0.274
(2)	*Gapdh*	1.682	*Gapdh*	0.482	*Gapdh*	0.21	1.16	1.9	*Gapdh*	0.359			*Tbp*	0.11	*Tbp*	0.326
(3)	*Tbp*	3	*Tbp*	0.511	*Tbp*	0.358	1.28	2.01	*Tbp*	0.423	*Tbp*	0.477	*Gapdh*	0.16	*Gapdh*	0.381

Stability values are calculated by using RefFinder Web-application (delta-C_t_ method, Bestkeeper, Normfinder, Genorm) with raw C_t_ values, all exported with the same threshold. In addition, Normfinder and geNorm were performed in R, where individual primer efficiency was taken into account

*SD* standard deviation, *CV* coefficient of variantion, *C*_*t*_ threshold cycle.

Even at different confluences it is shown that *Rpl22* reaches the best stability values. In contrast to the loaded samples, *Gapdh* performs better than *Tbp* in probes without mechanical loading. Again, Bestkeeper, Normfinder, geNorm and the delta C_t_ method show the same results (Table 3).

The results of Normfinder and geNorm gained with RefFinder were reviewed with the original Normfinder script for the statistical program R respectively with geNorm as a part of the NormqPCR package for R. In consideration of PCR primer efficiency in R, the results of RefFinder were confirmed for probes with loading compression. In contrast, outcome for probes with different confluences differ. In this case, *Rpl22* still wins the comparison, but *Tbp* is less stable compared to *Gapdh*.

### Influence of different cell confluences with and without loading compression in gene expression

**Figure 3:**
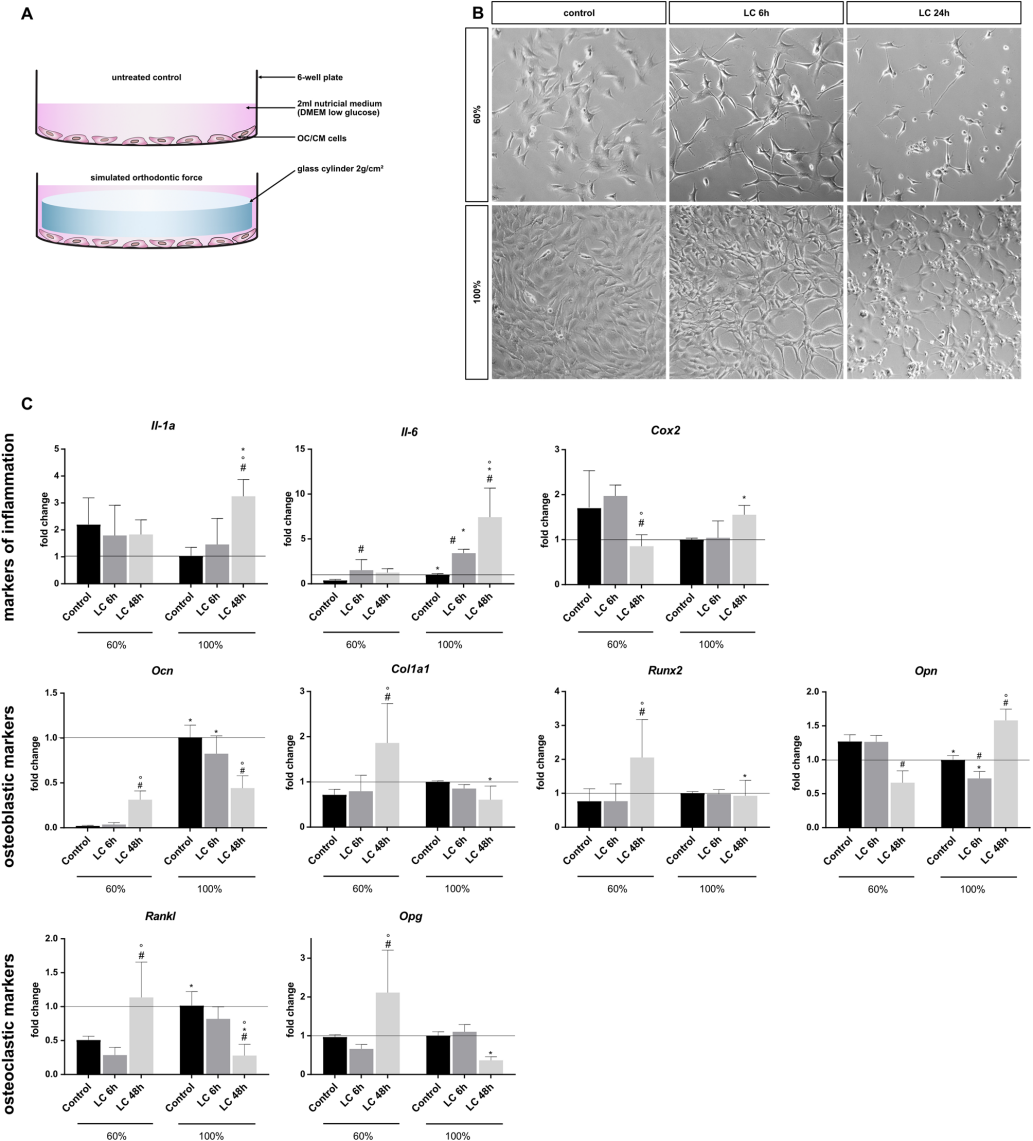
Influence of different cell confluences with and without loading compression in gene expression. (**A**) Illustration of the loading compression (LC) setup. Cell monolayer loaded with a sterile glass cylinder in 6-well plate to apply a static compression of 2 g/cm^2^. (**B**) Light microscopy photography of cells before loading (control) and before unloading (LC 6 h and LC 24 h). ×200 magnification, cropped, exposure adjustment and sharpening in Photoshop CC. (**C**) mRNA expression of selected markers of inflammation, osteoblastic and osteoclastic markers mean ± SD, n = 6 (two independent experiments in triplicate); normalization by ddC_t_ method to Rpl22 and control 100% statistical analysis with GraphPad Prism 7, two-way-ANOVA, p < 0.05, ^#^control vs. LC (loading compression); *60% vs. 100%; °LC 6 h vs. LC 48 h.

Using loaded and unloaded probes with 60% and 100% confluence, we aimed to analyse the effect of different cell confluences on gene expression of target genes know to be central regulators in periodontal remodeling. The target genes are divided in three groups: Markers of inflammation, osteoblastic markers and osteoclastic markers (Fig. 3).

According to our data the target gene expression differs under mechanical stress (LC) and at different confluences. Interleukin 6 (*Il-6*), Osteocalcin (*Ocn*) and Receptor Activator of NF-κB Ligand (*Rankl*) show a significant upregulation within the uncompressed controls with increased confluence. For Osteopontin (*Opn*), a significant downregulation could be observed. Interleukin 1a (*Il-1a*) and Cyclooxygenase 2 (*Cox2*) show a non-significant tendency of downregulation, while the basal expression of Collagen type 1a1 (*Col1a1*), Run-related transcription factor 2 (*Runx*2) and Osteoprotegerin (*Opg*) remains unaltered (Fig. 3).

Cells that are mechanically compressed at a confluence of 60% show different regulatory patterns for the investigated target genes. For example *Ocn, Col1a1, Rankl* and *Opg* show a significant upregulation after a loading compression time of 48 h. *Il-6* was only significantly upregulated after 6 h loading compression. There was a significant downregulation of *Cox2* and *Opn* after 48 h LC while the gene expression of *Il-1a* at 60% confluence remained unaltered.

At 100% confluence, the target gene expression was changed by the loading compression in a different manner. *Il-1a, Il-6, Cox2* and *Opn* were significantly upregulated after a LC of 48 h, *Il-6* even after 6 h LC while *Opn* was downregulated after 6 h LC time. *Ocn* showed a significant downregulation after 6 h and 48 h LC time, while *Col1a1, Runx22, Rankl* und *Opg* were significantly downregulated only after a LC time of 48 h.

Some markers showed reverse tendencies under loading compression at different cell densities. *Il-1a*, *Cox2* and *Opn* were downregulated when being compressed at 60% confluence while they are upregulated at 100%. *Ocn, Col1a1, Rankl, Opg* indicate opposite changes.

## Discussion

RT-qPCR is a commonly used method to study changes in gene expression due to its capacity of relative quantification of gene expression. It is always necessary to find at least one or better multiple individually tested reference genes for each cell type through experimental condition considering the error susceptibility and sensitivity of this method^35^.

Experiments with cells under mechanical compression require special caution in selection of reference genes because well-tried genes such as *Gapdh* and *ß-actin* do not perform well under these conditions^32,33,36^.

We have decided to test three reference genes: *Rpl22*, *Tbp* and *Gapdh*. *Gapdh* is a commonly known reference gene, that has already been used in first loading compression experiments with OC/CM cells^37^. For this reason, we included *Gapdh* in our selection despite its’ well known weakness in our test conditions. *Rpl22* performed best and *Tbp* performed very well as reference gene in loading compression experiments with periodontal ligament fibroblasts^34^. The selection of potential reference genes was based on the experiences with PDLF cells because of three facts: First, the spatial proximity of cementoblasts to PDLF. Second, we always use the same loading compression method. Third, PDL fibroblasts as well as cementoblasts are differentiated from the same progenitor cells^38^.

The self-designed primers are working well with satisfying specificity and efficiency. There are no side products detectable. Both methods for determining primer efficiency (dilution series and LineReg) show identical patterns: The *Tbp* primer has the lowest performance but it is still in the range of acceptable efficiency of 90–110%^39^.

In our analysis, *Gapdh* is the most regulated gene under mechanical stimulation. In contrast, *Rpl22* and *Tbp* gene expression seems to be less affected in our experiments. In particular *Rpl22* achieved the best results in all four algorithms for the use as a housekeeping gene according to the comparison of all potential reference genes. This matches with our previous publication about hPDLF^34^. Interestingly, the four algorithms, which show significant differences in their way of calculating stability, deliver identical results. These results were generated with RefFinder web application which offers an easy to use and fast way to analyze gene stability with four different algorithms in just one step. To obtain valid statements, these results have to be verified, because in some cases, RefFinder estimates stabilities differing from the original algorithms. These variation can be traced back to the input of raw C_t_ values without consideration of primer efficiencies^40^. For this reason, we compared the RefFinder results of Normfinder with the original Normfinder package and geNorm as part of the NormqPCR package in R. Even this control, where the primer efficiency was taken into account, shows no deviation from the previous results. This is a key argument for the reliability of our reference gene ranking for compression experiments.

Our second analysis in RefFinder with untreated cells at different confluences gives a hint of *Gapdh*s’ qualities as a classic reference gene. Without compression, *Gapdh* replaces *Tbp* at position 2 in the ranking. As before, *Rpl22* wins the comparison in all four algorithms and proves its’ usability in experiments without compressive stimulation. We confirmed these results by the calculation in R. However, calculated with primer efficiency, the ranking of *Tbp* and *Gapdh* shows up in reverse order.

Confluence plays a crucial role in the regulation of multiple target genes in loading compression experiments of OCCM cell lines. Exemplary markers of inflammation as well as markers of bone remodeling differ significantly in their mRNA expression between 60 and 100% confluence. Especially *Il-6* and *Ocn* show clearly shifted expression levels in the controls. This may correlate with the quantity of cell-junctions to neighboring cells, whose number changes with cell confluence (Fig. 3B). In addition, many markers (e.g. *Il-1a, Rankl, Ocn*) indicate inverted regulations under compression at variable confluences particularly after 48h strain, that can convert the outcome of a whole experiment. Therefore, a strict observation and documentation of cell confluence is essential for a meaningful and comparable result.

Based on our results, *Rpl22* is a recommended RT-qPCR reference gene for experiments with murine cementoblasts both under static compression and in analyses without a compressive stimulus. The combination of *Rpl22* and *Tbp* can improve the normalization, especially in loading experiments where *Tbp* scores clearly better than *Gapdh*. The use of *Rpl22* as reference gene will help in future investigations to discover the role of cementoblasts in orthodontic tooth movement and their functional distinction from fibroblasts. Cell confluence is a not neglectable aspect for getting reliable results in experiments with cementoblasts. A conscientious monitoring should be a part of experimental quality assurance.

## Material and methods

### Cell culture

Immortalized murine osteocalcin expressing cementoblasts (OC/CM), friendly provided by Prof. Somerman^24^, were cultured in DMEM low glucose (1 g/L) (Gibco, USA), 10% FCS (Gibco, USA), 100 units/mL of penicillin and 100 µg/mL of streptomycin (Gibco, USA) in cell culture plates under normal cell culture conditions (37 °C, 5% CO_2_, water saturated). Cells were trypsinized, centrifuged at 350*g*, quantified using Counting Chamber “Neubauer improved” and different numbers of cells were seeded in 6-well plates.

### Amplification, primer efficiency and validation

Primer specificity was controlled by 2 methods. First, a melting curve analysis was performed after RT-qPCR cycles. Second, the PCR products were analyzed by agarose gel electrophoresis. For this, RT-qPCR products (10 µL) were mixed with 4× loading dye (0.25% (w/v) bromophenol blue, 30% glycerol, 10 mM tris pH7) and loaded on a 2% agarose gel, which was prepared with GelRed Nucleic Acid Gel Stain (Biotium, USA). Amplification products were separated parallel to a 100 bp DNA ladder (Thermo Fisher Scientific, USA) at 120 V for 60 min in TAE buffer. Fluorescent bands were visualized by the ChemiDoc MP Imaging System (BioRad, USA).

Primer efficiency was determined with a 4 step log_10_ dilution series (100/10/1/0.1 ng/µL cDNA concentration of untreated cells). Two technical replicates per dilution level were used in 5 (*Rpl22* & *Tbp*) respectively 4 (*Gapdh*) independent experiments. Standard curves were created by linear regression of the resulting C_t_ values with the relative cDNA dilution. Basing on the slope of each standard curve, primer efficiency was calculated with $E=100*({10}^{-\frac{1}{slope}}-1)$. In addition, the amplification efficiency of each well (n = 50) was checked up by LineReg^41,42^ (https://www.medischebiologie.nl/files/).

### Compression experiments

The compression method, initially developed by Kanzaki et al.^26^ has already been used with cementoblasts^12,13,37^. According to this, cells were compressed with massive glass cylinders (2 g/cm^2^) in 6-well-plates for 3; 6; 12; 24 and 48 h. The loading times correspond to the time periods established in hPDLF experiments^1^. Before use, the glass cylinders were washed in deionized water, ethanol (70%) and finally autoclaved.

The pressure level used in the present investigation was 0.02 N/cm^2^ (similar to 2 g/cm^2^) and has been adapted from in vivo conditions in the periodontal ligament during orthodontic tooth movement in order to simulate mechanical loading in vitro with periodontal ligament fibroblasts^1,43,44^ and with cementoblast cells^13^.

### Isolation and purification of RNA

For RNA-isolation cells in each well were first washed with 2 mL phosphate-buffered saline (Gibco) and then harvested with 0.5 mL TRIzol™ Reagent (Thermo Fisher Scientific, USA), two wells were pooled. This leads to biological triplicates for each condition. After isolation, according to the manufacturer’s instructions, the RNA yield of each sample was verified photometrically at 280 nm and 260 nm (Nanodrop One™, Thermo Fisher Scientific, USA). Afterwards RNA purification was performed with RNeasy Mini Kit (Qiagen, Germany) following the producers’ protocol including an on-column DNA digestion (RNase-Free DNase, Qiagen, Germany). In order to control the success of the purification and to ensure a uniform cDNA synthesis, each sample was measured again (Nanodrop One™).

### Quantitative realtime-PCR analysis (RT-qPCR)

The RNA was transcribed into cDNA (SuperScript III RT, Thermo Fisher Scientific, USA). Basing upon the measurement after RNA purification, the final concentration was 25 ng/µL. All steps from RNA isolation to cDNA synthesis were performed in parallel for all samples of each experiment in order to avoid experimental variations.

RT-qPCR was performed in technical duplicates using 2.5 ng/µL cDNA in each reaction and a primer concentration of 0.5 µM. The qTower^3^ (Analytik Jena, Germany), High Green Mastermix (Thermo Fisher Scientific, USA), qPCR-Soft 3 (Analytik Jena, Germany) and self-designed intron spanning primers were used (Eurofins, Luxembourg). Primers were designed by using Primer-BLAST (NCBI, USA) followed by a PCR-Check (Eurofins Oligo Analyse Tool, Luxembourg) to ensure in silico qPCR specificity. Our criteria were length ca 20 bp, annealing temperature 60 °C, max product length 200 bp, intron spanning, covering possible transcript variants. The RT-qPCR protocol included an initial step of 50 °C for 2 min, 95 °C for 10 min followed by 40 cycles of 95 °C/15 s, 60 °C/30 s and 72 °C/30 s. A step of 95 °C for 15 s forms the transition to melting curve analysis (60–95 °C).

### Data analysis

Statistical analysis regarding reference gene stability was performed by using RefFinder^45^ Web-application including the following algorithms: GeNorm^46^, Normfinder^47^, Bestkeeper^48^ and comparative delta-C_t_ method^49^. RefFinder analyses raw C_t_-values which were exported from qPCRsoft 4.0 (Analytik Jena, Germany) with an automatic threshold. The C_t_ determines the number of RT-qPCR cycles required for the fluorescence to exceed a certain, defined detection threshold and shows an inverse correlation with the amount of cDNA template of the target gene sequence^49^. The highest automatically determined threshold was manually applied to all RT-qPCR plates across all experiments to ensure a clean separation between background noise and signal. This guaranteed that comparable C_t_ values could be generated for further analysis. Next, the mean value was determined from technical duplicates. These mean values from 4 independent experiments were combined into one data set for each gene.

Based on these data sets, two final data sets were generated. One contained exclusively control samples of different confluence while the other contained both loaded and control samples. Consequently, some control samples are part of both final data sets.

GeNorm performs a pairwise expression ratio under all control genes. The calculated stability value M is basing on average pairwise expression variations. As a result, genes with the lowest M value have the most stable expression^46^.

NormFinder was used to determine and combine expression variations of reference genes between the different groups (loaded/unloaded and 60%/100% confluence) and within each group to obtain a stability value^47^.

With BestKeeper we were able to determines the ‘optimal’ reference gene employing the pair-wise correlation analysis of all pairs of candidate genes. The geometric mean of the ‘best’ suited ones was calculated^48^.

The delta C_t_ method compares the relative expression of 'pairs of genes' within each sample. If the ΔC_t_ value between the two genes remains constant when analysed in different samples, it means either both genes are stably expressed among those samples, or co-regulated (here we assume the stability of both genes). If the delta-C_t_ changes, one or both genes are variably expressed^49^. The lowest value always implies the most stable gene expression in all four algorithms. Respecting the fact that RefFinder uses algorithms differ from the original without any possibility to take into account the PCR efficiency^40^, we also analyzed the dataset with the original algorithms in R. For this, C_t_ values were brought into linear scale (RQ) by RQ = E^−(minCt − sampleCt)^^40^ (E = primer efficiency basing on dilution series; see Table 2). Finally, these RQ values were processed with Normfinder in the statistic software R and with geNorm (part of the NormqPCR package^50^) in R.

### Statistical analysis

Data were analyzed using a two-way analysis of variance (ANOVA) with post hoc Tukey test (Prism version 8.1.0; GraphPad Software), where *p <* 0.05 was considered statistically significant. Graphs (Fig. 3) show mean values (n = 6) ± standard deviation (SD).

## Supplementary information

Supplementary Data 1
