# Author Correction: Selection and validation of reference genes by RT-qPCR for murine cementoblasts in mechanical loading experiments simulating orthodontic forces in vitro

**DOI:** 10.1038/s41598-021-82274-5

**Published:** 2021-02-17

**Authors:** Christian Niederau, Rogerio B. Craveiro, Irma Azraq, Julia Brockhaus, Asisa Bastian, Christian Kirschneck, Michael Wolf

**Affiliations:** 1 Department of Orthodontics, Dental Clinic, University of Aachen, Pauwelsstr. 30, 52074 Aachen, Germany; 2 Department of Orthodontics, University Medical Centre of Regensburg, Regensburg, Germany

Correction to: *Scientific Reports* 10.1038/s41598-020-67449-w, published online on 02 July 2020

The Author Contributions section in this Article is incorrect.

“C.N. and R.B.C. conceived the idea of the study and the study design. A.B. designed and C.N. and I.A. validated the used primer pairs. C.N., R. B. C., I.A., J.B. and M.W. contributed to discussion and study design. C.N. and I.A. conducted the experiments. C.K., C.N. and R.B.C. analysed the results. C.N. and I.A. wrote the manuscript and created the figures and tables. All authors reviewed the manuscript.”

should read:

“C.N. and R.B.C. conceived the idea of the study and the study design. A.B. designed and C.N. and I.A. validated the used primer pairs. C.N., R. B. C., I.A., J.B. and M.W. contributed to discussion and study design. C.N. and I.A. conducted the experiments. C.K., C.N. and R.B.C. analysed the results. C.N. wrote the manuscript and created the figures/tables. I.A. corrected the manuscript. All authors reviewed the manuscript.”

