# Supplementary material for: Selection and validation of reference genes by RT-qPCR for murine cementoblasts in mechanical loading experiments simulating orthodontic forces in vitro
Source: Sci Rep. 2020 Jul 2;10:10893. doi: 10.1038/s41598-020-67449-w (PMC7331740; doi:10.1038/s41598-020-67449-w)
Supplement: Supplementary file 1 — Supplementary Data 1 [file 41598_2020_67449_MOESM1_ESM.pdf]

Supplementary Data 1

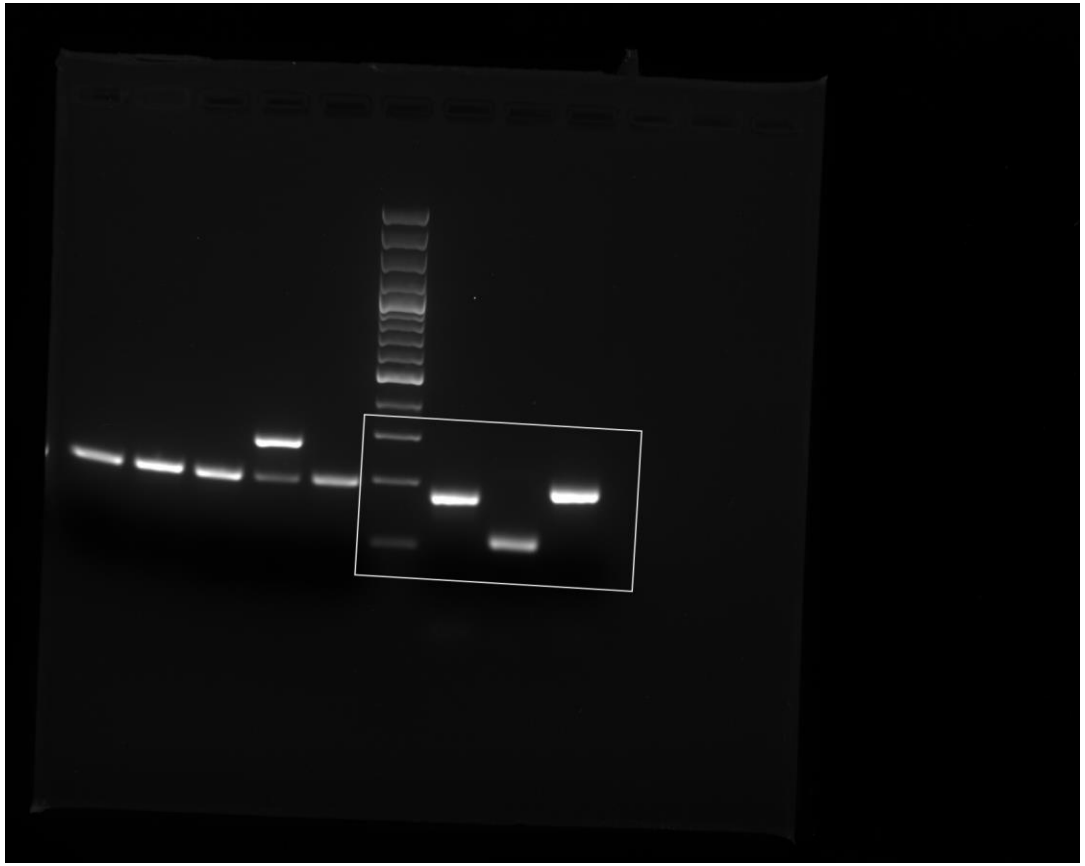

DNA gel with cropping marks

Non edited image of 2% agarose gel with RT-qPCR products separated by gel electrophoresis. The white frame shows cropping marks of the definitive figure 1B.
